# A phylogenetics and variant calling pipeline to support SARS-CoV-2 genomic epidemiology in the UK

**DOI:** 10.1093/ve/veae083

**Published:** 2024-10-17

**Authors:** Rachel Colquhoun, Áine O’Toole, Verity Hill, J T McCrone, Xiaoyu Yu, Samuel M Nicholls, Radoslaw Poplawski, Thomas Whalley, Natalie Groves, Nicholas Ellaby, Nick Loman, Tom Connor, Andrew Rambaut

**Affiliations:** Institute of Ecology and Evolution, University of Edinburgh, Ashworth Laboratories, Charlotte Auerbach Rd, Edinburgh EH9 3FL, United Kingdom; Institute of Ecology and Evolution, University of Edinburgh, Ashworth Laboratories, Charlotte Auerbach Rd, Edinburgh EH9 3FL, United Kingdom; Institute of Ecology and Evolution, University of Edinburgh, Ashworth Laboratories, Charlotte Auerbach Rd, Edinburgh EH9 3FL, United Kingdom; Department of Epidemiology of Microbial Diseases, Yale School of Public Health, 60 College St, New Haven, CT 06510, United States; Institute of Ecology and Evolution, University of Edinburgh, Ashworth Laboratories, Charlotte Auerbach Rd, Edinburgh EH9 3FL, United Kingdom; Vaccine and Infectious Disease Division, Fred Hutchinson Cancer Center, 1100 Fairview Ave. N., Seattle, WA 98109-1024, United States; Institute of Ecology and Evolution, University of Edinburgh, Ashworth Laboratories, Charlotte Auerbach Rd, Edinburgh EH9 3FL, United Kingdom; Institute of Microbiology and Infection, University of Birmingham, School of Biosciences, Birmingham B15 2TT, United Kingdom; Institute of Microbiology and Infection, University of Birmingham, School of Biosciences, Birmingham B15 2TT, United Kingdom; School of Biosciences, Cardiff University, Sir Martin Evans Building, Museum Avenue, Cardiff, Wales CF10 3AX, United Kingdom; TARZET Division, UK Health Security Agency, 10 South Colonnade, Canary Wharf, London E14 4PU, United Kingdom; TARZET Division, UK Health Security Agency, 10 South Colonnade, Canary Wharf, London E14 4PU, United Kingdom; Institute of Microbiology and Infection, University of Birmingham, School of Biosciences, Birmingham B15 2TT, United Kingdom; School of Biosciences, Cardiff University, Sir Martin Evans Building, Museum Avenue, Cardiff, Wales CF10 3AX, United Kingdom; Pathogen Genomics Unit, Public Health Wales, Number 2 Capital Quarter, Tyndall St., Cardiff CF10 4BZ, United Kingdom; Institute of Ecology and Evolution, University of Edinburgh, Ashworth Laboratories, Charlotte Auerbach Rd, Edinburgh EH9 3FL, United Kingdom

**Keywords:** SARS-CoV-2, genomic surveillance, genomic epidemiology, phylogenetics, software

## Abstract

In response to the escalating SARS-CoV-2 pandemic, in March 2020 the COVID-19 Genomics UK (COG-UK) consortium was established to enable national-scale genomic surveillance in the UK. By the end of 2020, 49% of all SARS-CoV-2 genome sequences globally had been generated as part of the COG-UK programme, and to date, this system has generated >3 million SARS-CoV-2 genomes. Rapidly and reliably analysing this unprecedented number of genomes was an enormous challenge. To fulfil this need and to inform public health decision-making, we developed a centralized pipeline that performs quality control, alignment, and variant calling and provides the global phylogenetic context of sequences. We present this pipeline and describe how we tailored it as the pandemic progressed to scale with the increasing amounts of data and to provide the most relevant analyses on a daily basis.

## Introduction

In the decade prior to 2020, viral genomic epidemiology emerged as a dynamic and rapidly evolving field. Phylogenetic analysis was used to infer the origins and diversity of HIV (Sharp and Hahn 2011) and influenza A virus, including during the 2009 swine flu epidemic (Smith et al. 2009). The decreasing cost of sequencing allowed it to be applied further to ‘large-scale’ datasets to infer transmission dynamics and influence public health decisions, first during the 2013–16 West African Ebola epidemic (Gire et al. 2014, Mate et al. 2015), and for each major epidemic since [Zika (Faria et al. 2017, Grubaugh et al. 2017), Middle East respiratory syndrome (Sabir et al. 2016), and Ebola in DRC (Kinganda-Lusamaki et al. 2021)]. Concurrently, visualization tools like Nextstrain (Hadfield et al. 2018) had been developed to enable interactive tracking of viral evolution. When the SARS-CoV-2 pandemic started, a global sequencing effort provided an unprecedented opportunity to use genomic surveillance to inform the public health response.

In March 2020, the COVID-19 Genomics UK (COG-UK) consortium was set up to provide a framework for national-scale, rapid whole-genome sequencing of SARS-CoV-2 within the UK in order to understand viral transmission and evolution and inform public health responses in real-time. This national partnership included the four UK Public Health Agencies, National Health Service (NHS) organizations, regional sequencing centres, and academic partners (COVID 2020). The data generation arm of the consortium operated as a decentralized network of labs, in both healthcare and academic settings, collecting and genome sequencing SARS-CoV-2 samples. The genome sequences and associated metadata were then submitted to a central platform, CLIMB-COVID (Nicholls et al. 2021), where it was collected into a single canonical dataset.

A genome sequence without appropriate associated metadata is of limited use, so we quickly established a minimal metadata standard that could contextualize genomes in time and space. This minimum standard facilitated informative analysis of the data while limiting the burden of participation. It required a collection date, or the date a sample was received by a lab, country- and county-level geographic information (administration levels 1 and 2 in the UK), and a record of whether the sample was collected as part of a random surveillance strategy or a targeted outbreak analysis. Additional metadata fields could be supplied and, in practice, this has resulted in a rich and detailed dataset with a consistency of useful information that has been invaluable to consortium members across the UK. This level of private metadata sharing was only possible within a controlled UK-based shared computing environment.

To interpret any new genome sequence, it needs to be compared to and contextualized within the recent local and global diversity, most commonly in the form of a phylogenetic tree. Most phylogenetic methods were not developed with this unprecedented amount of data in mind and require large computational resources that scale poorly with increasing numbers of sequences. To overcome this, we needed to develop an analysis pipeline that processed this dataset centrally on a daily basis, performing alignment and variant calling, and amalgamated the COG-UK data with publicly available global sequences to provide the phylogenetic context. Outputs of this pipeline were made available within the consortium and provided interpretable results for both local NHS health protection teams and the UK public health agencies. They were consumed by public data explorers, including COG-UK-ME (Wright et al. 2022), Microreact (Argimón et al. 2016), and the UCSC Genome Browser phylogenetic tree (McBroome et al. 2021), and provided the basis of individual local outbreak investigations using CIVET (O’Toole et al. 2022).

## Results

**Figure 1.**
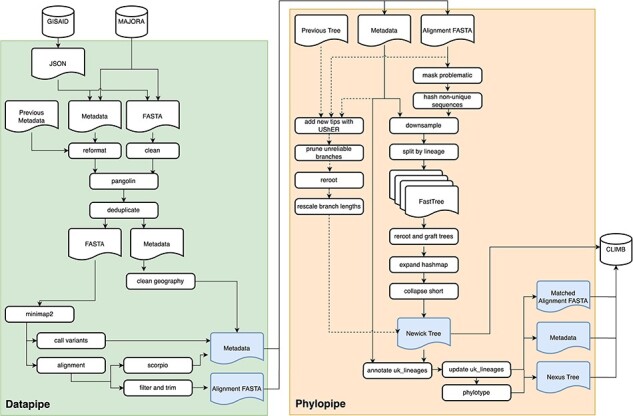
An overview of the analysis pipeline, split into two workflows represented by Datapipe and Phylopipe. Blue boxes represent the main outputs, with specific subsets and combinations of these files published on CLIMB-COVID. Datapipe accepts incoming FASTA and metadata TSV files generated by the ELAN pipeline (Nicholls et al. 2021) and performs initial QC of sequences and metadata, lineage assignment, alignment and variant calling, deduplication, filtering and geography cleaning. It combines the dataset with sequences from GISAID which have been processed similarly. Phylopipe consumes datapipe output and either constructs a new phylogenetic tree by grafting together subtrees constructed with FastTree (Price et al. 2010), or adds to an existing tree with UShER (Turakhia et al. 2021). The resulting newick tree is annotated, and phylogenetic summary information is inferred including uk_lineages and phylotypes.

The analysis pipeline that supported the UK efforts is divided into two workflows (‘Datapipe’ and ‘Phylopipe’, Fig. 1) written in the nextflow (Di Tommaso et al. 2017) workflow language. Datapipe (https://github.com/COG-UK/datapipe) performs alignment and variant calling, and Phylopipe (https://github.com/virus-evolution/phylopipe) constructs the phylogenetic tree. These replace the original single snakemake (Mölder et al. 2021) pipeline, grapevine (https://github.com/COG-UK/grapevine), which performed both workflows until February 2021. A high-level overview of each pipeline is provided here along with the design decisions, with further details provided in the Supplementary material.

### Datapipe

This consumes the FASTA and metadata TSV file(s) generated by the ELAN pipeline (https://github.com/SamStudio8/elan-nextflow/) and runs variant calling and alignment. First, we clean non-nucleotide symbols from sequences and reformat metadata. New samples are assigned a Pango lineage with pangolin (O’Toole et al. 2021b), with all samples reassigned if the underlying model has been updated. Sequences are deduplicated if they correspond to the same biological isolate and background/global sequences with the same sample_name are deduplicated by date.

A trimmed FASTA alignment is generated using minimap v2.17 (Li 2018) to pairwise align each sequence to Wuhan-Hu-1 (GenBank: MN908947.3; https://www.ncbi.nlm.nih.gov/nuccore/MN908947) and gofasta (Jackson 2022) to combine and mask 5ʹ and 3ʹ ends. Insertions relative to the reference are discarded. All nucleotide mutations, insertions, and deletions with respect to the reference are noted in the metadata. Sequences that consist of >5% unknown sites (‘N’) per genome after mapping are discarded. Geographical metadata is cleaned (https://github.com/COG-UK/geography_cleaning) and the UK dataset is combined with all non-UK sequences from Global Initiative on Sharing All Influenza Data (GISAID) (Elbe and Buckland-Merrett 2017), which we process similarly on a weekly basis.

Finally, subsets of the metadata and alignment files are published using a configuration JSON. This includes outputs with sensitive data removed that can be made available to the public via the consortium website and s3 buckets (https://www.cogconsortium.uk/), as well as specific subsets that are consumed by data explorers including the COG-UK Mutation Explorer (Wright et al. 2022) and CIVET (O’Toole et al. 2022).

#### Design decisions

Pango data releases were regular for most of the pandemic and often resulted in the most recent sequences being reclassified as new lineages were defined. For this reason, we added a step to the pipeline to check if a new version had been released, in which case we would reclassify all sequences.

The very first implementations of the pipeline used Multiple Sequence Alignment; however, this approach scales quadratically with the number of sequences and a pairwise approach using gofasta was necessary from very early in 2020. The alignment was trimmed with the 5ʹ and 3ʹ ends masked with N’s as these regions were typically more error prone.

While outputs were initially hardcoded, we later started generating outputs using a JSON ‘recipe’ file. This allowed different subsets of the sequences and metadata to be defined easily as the downstream requirements for analysis changed over time.

### Phylopipe

This consumes the FASTA and metadata CSV file(s) generated by the datapipe pipeline and either constructs a phylogenetic tree using FastTree (Price et al. 2010) or adds to an existing tree with UShER (Turakhia et al. 2021).

First, globally problematic sites (flagged homoplasic sites, sites with mutations arising multiple times across the canonical global phylogeny, and nanopore adaptor sites) are masked in the combined (UK and global) alignment, and sequences with too many ambiguous bases are excluded.

To construct a new phylogenetic tree, nonunique sequences are hashed to a single representative. Optionally sequences are further reduced by heavy downsampling by date and lineage diversity. The reduced alignment is split based on Pango lineage assignment into six large distinct sublineages and subtrees are built independently for each using the Jukes–Cantor model (Jukes and Cantor 1969) in FastTree (Price et al. 2010) v2.1.10 (double precision). The resulting subtrees are rooted and grafted together by attaching the root of incoming trees to the same taxon’s tip in the parent tree, and nonunique sequences are inserted alongside their representative. Branch lengths less than 5E-6, which represent distances smaller than one SNP and result from ambiguities between sequences, are collapsed to 0.

UShER (Turakhia et al. 2021) is used to update this tree with additional sequences using maximum parsimony. Branches with >30 private mutations are pruned from the tree as artefactual, branch lengths are rescaled, and the tree is rerooted on Wuhan/WH04/2020.

The tips of the full tree are annotated with binary UK/non-UK trait information and fine scale uk_linages representing independent UK introductions from other countries.

The resulting annotated tree and metadata are disseminated to the consortium. Again specific outputs are published using a configuration JSON, including those for Microreact (Argimón et al. 2016).

#### Design decisions

As full tree construction typically scales worse than quadratically with the number of sequences, we implemented several steps to try and reduce the number of sequences considered by a tree-building algorithm. In the first case, we hashed nonunique sequences, including only a single representative sequence type during the tree-building step. These sequences were then inserted alongside their representative in the resulting tree. This approach is mirrored internally by some tree-building methods such as FastTree but not all. Secondly, we partitioned the sequences into groups based on their Pango lineage assignment, which we expected to represent well-defined subtrees with a clear out-group. When these groups are relatively evenly sized, this approach effectively divides the expected total time to construct the tree by the number of groups, with a much greater time saving if the subtrees are then constructed in parallel instead of consecutively. Finally, updating the tree with UShER allows only new sequences to be added in approximately linear time.

In addition to defining UK introductions, the subtree for each UK lineage was annotated with phylotypes by codifying the internal nodes of the tree, effectively representing parent/child/sibling phylogenetic relationships in metadata. This proved hugely beneficial to public health agencies as it enabled interpretation of phylogenetic relationships in a format that could be represented on reports without requiring tree visualization software or interpretation.

## Discussion

### Scaling with the pandemic

The pipelines described earlier have had to evolve considerably as the pandemic has progressed both in order to stay relevant to the questions being investigated by public health bodies and in order to continue to scale with exponentially growing levels of data.

#### A relevant resource

In the early phase of the pandemic, key questions on a national and local health level focused on quantifying the number of introductions into an area and assessing subsequent spread (Da Silva Filipe et al. 2020, Du Plessis L et al. 2021, Lycett et al. 2021). At this time, the pipeline automatically generated weekly reports that summarized the latest data at the national (UK-wide) level, within each of the four constituent countries of the UK (Wales, Scotland, Northern Ireland, and England), and at a further regional level corresponding to several of the COG-UK sequencing partners. These reports included case counts of individual lineages and estimates of the numbers of new introductions and subsequent spread based on uk_lineage information. As cases rose the outputs fed into the COG-UK coverage maps used by government and as more bespoke investigative reports were in demand, we added specific pipeline outputs to support local report generation using CIVET (O’Toole et al. 2022), in addition to those that already existed to support Microreact (Argimón et al. 2016).

Following the first lockdown and as the initial variants of concern (VOCs) emerged, the focus of investigations shifted to mutations, lineages, and constellations for VOCs and variants under investigation, rather than the previous focus on introductions. Relevant steps were added into the pipeline to type them and these classifications were fed into the COG-UK Mutation Explorer (Wright et al. 2022) and GRINCH (O’Toole et al. 2021a).

#### Timely results

The initial pipeline grapevine (https://github.com/COG-UK/grapevine) was written in Snakemake (Mölder et al. 2021). By January 2021, it became clear that the phylogenetics steps of the pipeline were becoming prohibitively slow. To enable the continued rapid dissemination of sequence data to the consortium, the pipeline was separated into a data processing pipeline (https://github.com/COG-UK/datapipe), which rapidly performed the initial alignment, variant calling, and lineage assignment steps, and a phylogenetics pipeline (https://github.com/cov-ert/phylopipe), which consumed the output of the data processing pipeline and performed the tree building and post-processing steps. This allowed the data processing pipeline to run reliably every day, while the phylogenetics pipeline was allowed to run less frequently.

During this rewrite, we moved from using Snakemake workflow language to Nextflow. This was motivated by the observation that the Nextflow workflow manager seemed better able to handle issues arising on the SLURM (Jette and Wickberg 2023) computing cluster resource manager due to the large resource requirements, for example when a node became unresponsive.

#### Scaling phylogenetic methods

The phylogenetic tree construction steps have also had to adapt considerably with the growth of global data. Initially, we used IQ-TREE to estimate the global phylogeny (Nguyen et al. 2015, Minh et al. 2020). Then, for speed we introduced a process of assigning sequences to three distinct lineages A, B, or B.1, estimating these trees independently with IQ-TREE and subsequently grafting together these subtrees to form our global tree. By June 2020, after a series of benchmarking experiments, we adopted FastTree (Price et al. 2010) as the inference engine for parsimony-based tree reconstruction and this method was sufficient for our needs for the rest of 2020, with new subtrees (representing emerging sublineages such as B.1.1) added when appropriate.

By January 2021, with the advent of VOCs leading to a global surge in SARS-CoV-2 genome sequence generation, the pipeline was again struggling to complete regularly and it was not possible to parallelize further with new subtree splits. As a result, in February we began downsampling the data before tree-building. We initially subset to the previous 6 months, but as scaling continued to be a problem, this was further restricted to 5 months, then 100 days, and finally to 30 days plus background data. Even with this heavy downsampling, construction of the split-grafted tree using FastTree at this time took >2 days, meaning an interim solution would be needed for ‘real-time’ analysis. As such, we introduced daily tree updates with UShER (Turakhia et al. 2021), with less frequent tree rebuilds as and when the daily tree became unwieldy or gained errors.

#### Surprising bottlenecks

Because of the sheer scale of numbers of genome sequences, every aspect of the pipeline has been evaluated for both time and memory efficiencies. Simple processes such as reading and manipulating FASTA and metadata CSV files became significant bottlenecks because of how large these files had become. We found that the highly optimized pandas (McKinney 2010) dataframe library required too much memory and had to replace it with a custom metadata reader module based on the DictReader class from the python csv module. This custom suite of utility functions (https://github.com/cov-ert/fastafunk) instead streamed the metadata file twice and used set manipulation in order to hold minimal data in memory.

During the rewrite from grapevine to datapipe and phylopipe, we moved from a system where rows/sequences filtered at each stage were removed from the relevant files to one where the metadata table remained complete, with a column tracing why any given sequence had been eliminated from output FASTA. While there would have been performance gains from reducing metadata size, in practice, this method can easily log information for members of the consortium to know exactly why their sequences were missing from final metadata tables without becoming a time-consuming tracing exercise for the pipeline maintainers.

#### Recommendations for next time

There are a number of design choices we would recommend for a centralized analysis pipeline in a future outbreak scenario. First, genomic epidemiological analysis depends heavily on being able to index between genome sequence data and metadata: filtering to a subset of sequences, applying some analysis, and updating the metadata table with the results. We recommend working from the start either with a custom database or with the most lightweight, optimized, and well-tested software libraries available. Secondly, we recommend using restricted metadata fields wherever possible (rather than free text) in order to remove an ongoing burden of maintenance. We also recommend that the first step in the pipeline is to check and clean sequences, sequence headers, and metadata, including nonunicode characters. Thirdly, we recommend building in sample traceability from the start so that it is easy to identify why a given sample may not have been retained in the final analysis steps. Finally, the success of this pipeline was in our ability to adapt it to the most pressing analysis questions. We recommend designing code to be as clean and modular as possible so that less relevant analysis steps can be removed and more relevant ones added over time while retaining a consistency of output for downstream tools.

### The impact of centralized phylogenetic analysis

The UK was one of the earliest countries to adopt a national genomic surveillance programme for SARS-CoV-2 (COVID 2020). After the emergence of the Alpha variant, many more countries began to use genomics for surveillance. Many surveillance approaches make use of Nextstrain (Hadfield et al. 2018) builds; however, these are heavily downsampled and only include a small subset of the data. Early in 2020, the sarscov2phylo public tree (Mansfield 2020) or later the daily updated UCSC tree (McBroome et al. 2021) provided a full global phylogeny of public data. However, the early and extensive genomic sequencing within the UK, the detailed private metadata collected with governance to be shared within the distributed network of data users, and a requirement for numerous custom downstream analyses based on the full global phylogeny, all contributed to the requirement for a local pipeline.

The real power of performing these analyses centrally was that all members of the consortium were able to access detailed analysis and relevant information about their submitted data without extensive bioinformatics knowledge or the requirement for large computational resources to build a phylogenetic tree.

At the government level, simple representations of the data including the number of lineages over time fed national dashboards. COG-UK reports also fed into SAGE meetings. At the Public Health Agency level, unpublished summaries formed the basis of national surveillance projects and informed responses (COG-UK). At the local or regional hospital level, the outputs enabled investigations of hospital-onset SARS-CoV-2 infections (Stirrup et al. 2021) such as with the CIVET tool (O’Toole et al. 2022) informing outbreak management and wider infection prevention and control measures. This tool directly accessed the output of initially grapevine and later datapipe and phylopipe. Some of the resolutions within these reports came from the uk_lineage and phylotype metadata fields. These provided a fine-scale text representation of the phylogenetic relationships between samples in the UK and global tree, which could be interpreted without specialist tree viewing software or bioinformatics expertise.

More generally, outputs from this centralized analysis pipeline were used in analyses to reveal the multiple introductions of SARS-CoV-2 from mainland Europe into Scotland in 2020 (Da Silva Filipe et al. 2020) and to show that the Alpha variant was associated with increased clinical severity (Pascall et al. 2023). They were used to identify and verify early recombinant genomes (Jackson et al. 2021) and to test lineage frequencies from wastewater surveillance sequencing by comparison with conventional surveillance sequencing in the same geographic location (Brunner et al. 2023). The phylogenetic trees were used to provide routine early tracking of emerging variants (Drake et al. 2024), investigate how genetic drift changes over time (Yu et al. 2024), and investigate the impact of viral mutations on recognition by T cells (De Silva et al. 2021). They also provided the means to select a targeted downsample for more in-depth analyses, such as into the emergence and growth of the SARS-CoV-2 Delta variant in the UK (McCrone et al. 2022).

Finally, at a public level, data explorers such as COG-UK-ME (Wright et al. 2022), Microreact (Argimón et al. 2016), GRINCH (O’Toole et al. 2021a), and the UCSC Genome Browser phylogenetic tree (McBroome et al. 2021) were able to ingest pipeline outputs and allow exploration of the vast data resource more widely. As a result, the outputs of this pipeline have fed into many applications and impacted analyses both within the UK and globally.

## Supplementary Material

veae083_Supp

## Data Availability

All codes are open-source and available under a GNU GPL-3.0 licence in the GitHub repositories https://github.com/COG-UK/datapipe and https://github.com/virus-evolution/phylopipe.
