# Supplementary material for: A phylogenetics and variant calling pipeline to support SARS-CoV-2 genomic epidemiology in the UK
Source: Virus Evol. 2024 Oct 17;10(1):veae083. doi: 10.1093/ve/veae083 (PMC11529618; doi:10.1093/ve/veae083)
Supplement: veae083_Supp [file veae083_supp.zip › Phylopipe Supplementary Material.docx]

Supplementary Material

## Detailed pipeline overview


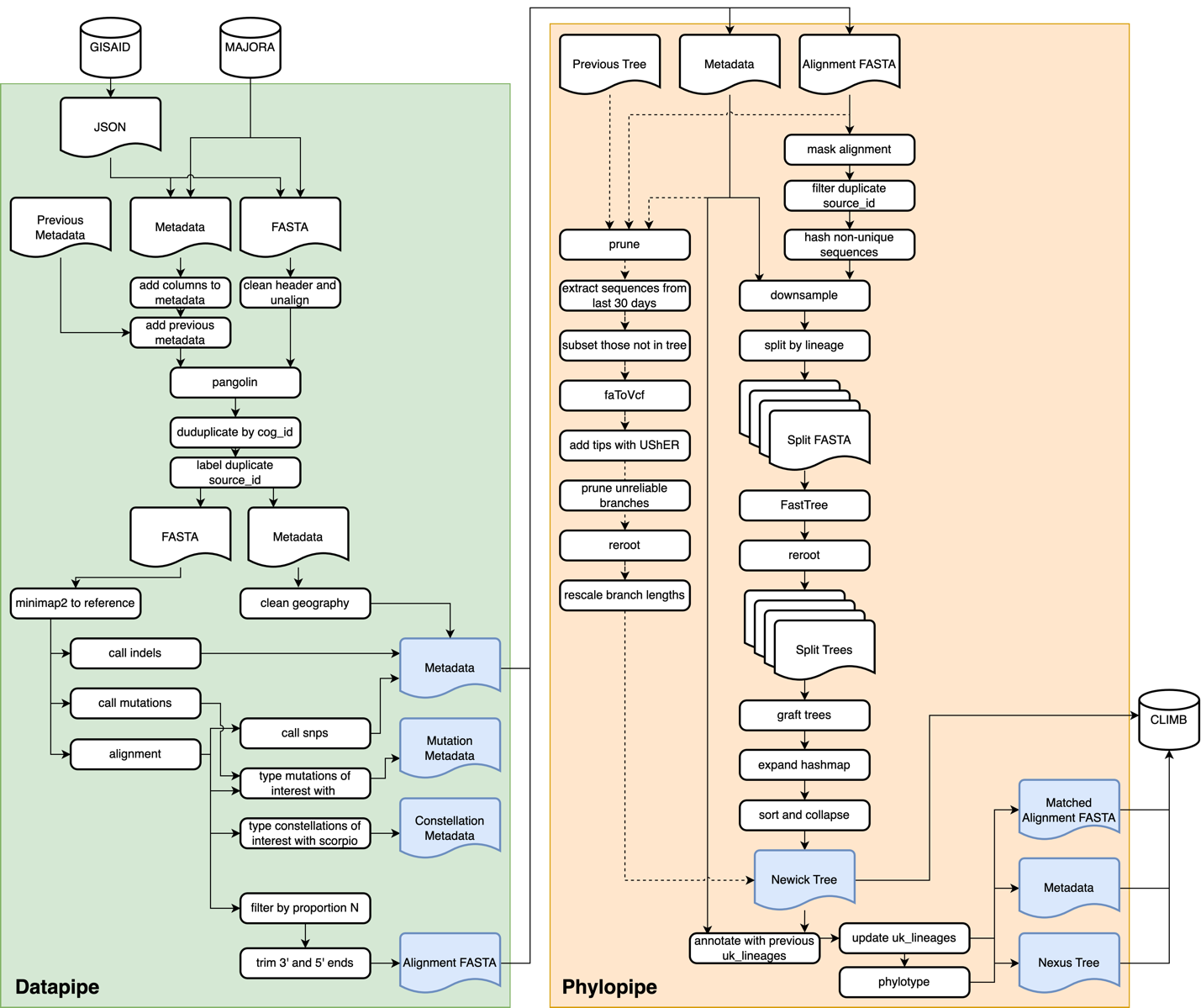


Supplementary Figure 1: A detailed overview of the analysis pipeline, split into two workflows represented by *Datapipe* and *Phylopipe*. Blue boxes represent the main outputs, with specific subsets and combinations of these files published on CLIMB-COVID. Datapipe accepts incoming FASTA and metadata TSV files generated by the ELAN pipeline [[11]](https://www.zotero.org/google-docs/?0NZclN) and performs initial QC of sequences and metadata, lineage assignment, alignment and variant calling, deduplication, filtering and geography cleaning. It combines the dataset with sequences from GISAID which have been processed similarly. Phylopipe consumes datapipe output and either constructs a new phylogenetic tree by grafting together subtrees constructed with FastTree [[16]](https://www.zotero.org/google-docs/?2hC4Ub), or adds to an existing tree with UShER [[17]](https://www.zotero.org/google-docs/?zhasGl). The resulting newick tree is annotated, and phylogenetic summary information is inferred including uk_lineages and phylotypes.

### Datapipe

This consumes the FASTA and metadata TSV file(s) generated by the ELAN pipeline (<https://github.com/SamStudio8/elan-nextflow/>) and runs variant calling and alignment. First we clean non-nucleotide symbols from sequences (de-aligning where necessary), and add to metadata using the information from across columns:

- sample_name is composed of the country (England/Scotland/Wales/Northern_Ireland), central_sample_id and year and represents a short informative name.
- sample_date is the collection_date if provided, and otherwise the received_date. At least one of these is required to submit metadata.
- epi_week and epi_day convert the sample_date to the pandemic-week and day in which it falls, with week commencing 2019-12-22 as week 0. This represents the week of the earliest sequenced genomes, although modelling suggests the pandemic began earlier.
- source_id combines root_biosample_source_id or biosample_source_id (with preference for root_biosample_source_id). Both represent samples from the same patient source, but are completed by different sequencing teams.
- is_pillar_2 is set if collection_pillar is specified as 2, or if central_sample_id has been generated by a known pillar 2 organisation. This is indicative of surveillance sequencing as opposed to targeted hospital sequencing.
- We add the GISAID and ENA accessions for samples that have been newly uploaded.
- We add the uk_lineage column from the previous phylopipe output.

New samples are assigned a Pango lineage with pangolin [[20]](https://www.zotero.org/google-docs/?1nhpjw), with all samples reassigned if the underlying model has been updated since the previous pipeline run. Sequences with the same central_sample_id (which represent the same extracted isolate) are deduplicated by (unmapped) proportion of ambiguous bases. Background/ global sequences with the same sample_name are deduplicated by date, keeping the earliest of duplicated sequences. Sequences with the same source_id represent longitudinal samples from the same patient, and are labelled for filtering by the phylogenetics pipeline with the earliest kept as representative.

Next, each sample is aligned to Wuhan-Hu-1 (GenBank: MN908947.3; <https://www.ncbi.nlm.nih.gov/nuccore/MN908947>) using minimap v2.17 [[21]](https://www.zotero.org/google-docs/?Hgj2mf) and the resulting SAM format file is converted to FASTA format using gofasta [[22]](https://www.zotero.org/google-docs/?eUMqOW), with the 5’ and 3’ UTRs of each sequence masked by Ns. Insertions relative to the reference are discarded from the final alignment, but are logged. All nucleotide mutations, insertions and deletions with respect to the reference are combined into a nucleotide_mutations column in the metadata and specific amino acid mutations and deletions of interest are typed. Sequences that consist of more than 5% unknown sites per complete genome after mapping are discarded. Geographical metadata is cleaned (<https://github.com/COG-UK/geography_cleaning>) and the UK dataset is combined with all non-UK sequences from GISAID [[23]](https://www.zotero.org/google-docs/?scTMTv), which we process similarly on a weekly basis.

Finally, subsets of the metadata and alignment files are published using a configuration JSON. This includes outputs with sensitive data removed that can be made available to the public via the consortium website and s3 buckets (https://www.cogconsortium.uk/), as well as specific subsets which are consumed by data explorers including the COG-UK Mutation Explorer [[12]](https://www.zotero.org/google-docs/?e7mJH1), and CIVET [[15]](https://www.zotero.org/google-docs/?jrsYlo).

### Phylopipe

This consumes the FASTA and metadata CSV file(s) generated by the datapipe pipeline and either constructs a phylogenetic tree using FastTree [[16]](https://www.zotero.org/google-docs/?PcXW2k), or adds to an existing tree with UShER [[17]](https://www.zotero.org/google-docs/?HMWPzF).

Globally problematic sites (flagged homoplasic sites, sites with mutations arising multiple times across the canonical global phylogeny, and nanopore adaptor sites) are masked in the combined (UK and global) alignment, and the UK longitudinal duplicates labelled during datapipe are removed. Sequences with ambiguous bases at a further set of flagged sites are removed to improve the candidate pool for downsampling. FASTA headers and the corresponding sample_name column in the metadata are cleaned of special characters that may cause errors with downstream phylogenetic tools.

To construct a new phylogenetic tree, first non-unique sequences are hashed to a single representative to reduce the computational load during initial tree construction. Since February 2021, sequences were further reduced by heavy downsampling, including only sequences from the most recent 2 months and a skeleton of background samples selected to cover diversity. The reduced alignment is split based on PANGO lineage assignment into 6 large distinct sub-lineages and sub-trees are built independently for each using the Jukes-Cantor model in FastTree [[16]](https://www.zotero.org/google-docs/?aEDHct) v2.1.10 (double precision). This splitting relieves some of the burden on tree building. The resulting sub-trees are rooted and grafted together by attaching the root of incoming trees to the same taxon’s tip in the parent tree, and non-unique sequences are inserted alongside their representative. Branch lengths less than 5E-6, which represent distances smaller than one SNP and result from ambiguities between sequences, are collapsed to 0. UShER [[17]](https://www.zotero.org/google-docs/?Mdg1tg) is used to update this tree with additional sequences using maximum parsimony. Branches with more than 30 private mutations are pruned from the tree as artifactual, branch lengths rescaled and the tree rerooted on Wuhan/WH04/2020.

The tips of the full tree are annotated with binary UK/non-UK trait information. Fine scale uk_lineages then are assigned to represent independent UK introductions from other countries. UK lineages based on the previous phylogenetic tree are labelled, and these are reviewed based on the new tree. The subtree for each UK lineage is then annotated with phylotypes by codifying the internal nodes of the tree. This effectively represents local phylogenetic relationships in a metadata table, allowing easy interpretation of parent/child relationships without requiring tree visualisation software.

The resulting annotated tree and metadata are disseminated to the consortium. Again specific outputs are published using a configuration JSON, including those for Microreact [[13]](https://www.zotero.org/google-docs/?Rfo8k0).
